# Spatial variation in sexual size dimorphism and mating associations in the color polymorphic Eastern Red-Backed Salamander (*Plethodon cinereus*)

**DOI:** 10.1007/s00442-025-05826-w

**Published:** 2025-11-05

**Authors:** Maggie M. Hantak, Olivia L. Brooks, Kyle M. Brooks, Carl D. Anthony, Cari-Ann M. Hickerson, Kelly A. Williams, Shawn R. Kuchta

**Affiliations:** 1 Department of Biology, University of Dayton, Dayton, OH 45469 USA; 2 Department of Biological Sciences, Virginia Polytechnic Institute and State University, Blacksburg, VA 24061 USA; 3 Department of Biological Sciences, Ohio Center for Ecological and Evolutionary Studies, Ohio University, Athens, OH 45701 USA; 4 Department of Biology, John Carroll University, University Heights, OH 44118 USA

**Keywords:** Amphibian, Assortative mating, Color morphs, Mate choice, Plethodontidae

## Abstract

Sexual selection due to mate preference for certain traits can maintain phenotypic diversity within populations and species. In taxa with discrete phenotypes, such as color polymorphic species, assortative mating may lead to disruptive selection and sympatric divergence, yet how such interactions vary over species' ranges remains poorly understood. To address this shortcoming, we examined spatial patterns of sexual size dimorphism and presumptive male–female mating pairs based on body size and coloration in the color polymorphic Eastern Red-backed Salamander, *Plethodon cinereus*. This species exhibits two common color morphs, striped and unstriped, that also differ in other elements of their biology, including ecology, behavior, and morphology. Across six populations that vary in color morph frequency, we predicted each site would show female-biased sexual size dimorphism and a positive body size relationship between presumptive mating pairs. In polymorphic sites, we also predicted that morphs would demonstrate color-based assortative mating, with larger body size as an associated trait. We found female-biased sexual size dimorphism in three of six study sites, while a positive body size relationship between male and female pairs was documented in four populations, and no evidence of male–female associations by color and body size was found in polymorphic populations. The spatial variation across all of our study axes demonstrates the importance of geographic context in shaping sexual selection dynamics and patterns of local adaptation.

**Supplementary Information:**

The online version contains supplementary material available at 10.1007/s00442-025-05826-w.

## Introduction

Sexual selection due to mate preference for specific traits can have a profound influence on phenotypic diversity within species (Andersson 1994; Debelle et al. 2014; Mendelson and Safran 2021). Positive assortative mating—the non-random mating of phenotypically similar individuals—is important because it can result in disruptive selection on phenotypic traits, or under some conditions be a precursor to the evolution of reproductive isolation between distinct forms (Foote and Larkin 1988; Dieckmann and Doebeli 1999; Elmer et al. 2009; Servedio and Boughman 2017). Some past studies have considered a spatial context when examining patterns of assortative mating (e.g., Scott 2004; Maan and Cummings 2008; Dreher and Pröhl 2014), but most work has focused on single populations, even though environmental factors such as microhabitat, resource availability, and predation pressures vary across regions, which can lead to divergent selective pressures that shape mate preferences in different areas (Schluter 2000). For example, Seehausen et al. (1997) demonstrated that eutrophication-induced turbidity in Lake Victoria relaxed sexual selection on male coloration in cichlid fish, leading to reduced reproductive isolation compared to clearer areas of the lake. Spatial differences in mate preferences may, therefore, have a large impact on geographic patterns of phenotypic variation and reproductive isolation (Huyghe et al. 2010; Moura et al. 2021). As such, examining mating interactions across space can provide information on the maintenance of phenotypic and genetic variation, especially within widespread species.

In addition to biotic and abiotic variability across a species’ range, the presence of multiple discrete phenotypes may also influence mating interactions. Color polymorphic species, in which two or more distinct, genetically determined color morphs coexist within a single interbreeding population, provide a valuable system to study the processes contributing to the evolution and maintenance of phenotypic variation and potentially reproductive isolation (Ford 1945; Gray and McKinnon 2007). Morphs are not simple color-pattern variants, but are shaped by multivariate diversifying selection and are comprised of alternative sets of co-adapted traits (Sinervo and Svensson 2002), which may play a key role in promoting ecological divergence (McKinnon and Pierotti 2010). Morphs may also lead to species formation when a population loses a morph, or fixes on a single morph, due to the release of genetic and selective constraints resulting in rapid evolutionary change (West-Eberhard 1986; Corl et al. 2010; Waldron et al. 2025a). How and why color morphs are maintained is a dynamic question in evolutionary biology, and understanding the role of mating interactions in polymorphic systems can be a useful step in identifying how morphic variation is maintained (McLean and Stuart-Fox 2014).

**Fig. 1:**
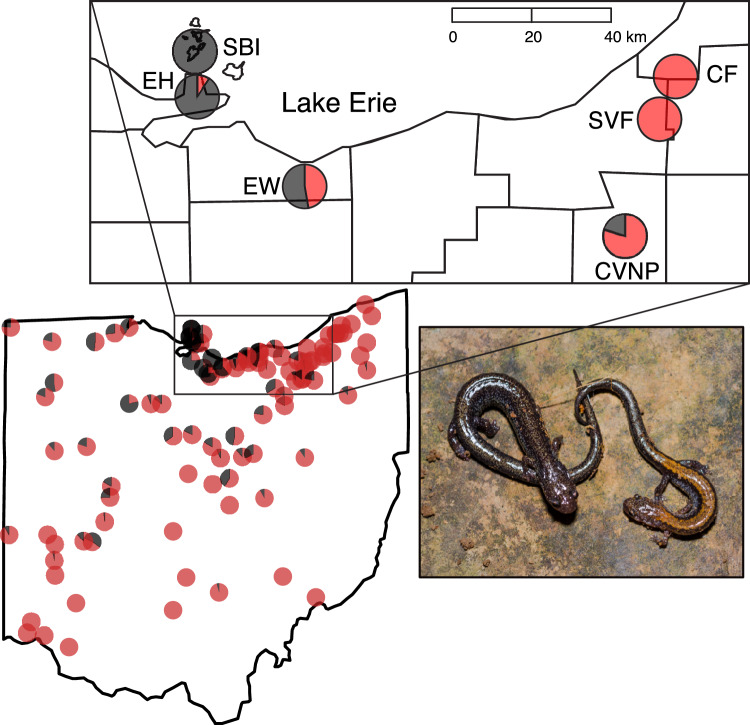
Map of color morph frequencies (black = unstriped, red = striped) in Ohio. Study sites: Chapin Forest Reservation (CF; 100% striped), Cuyahoga Valley National Park (CVNP; 80% striped), East Harbor State Park (EH; 8% striped), Edison Woods Reservation (EW; 45% striped), South Bass Island (SBI; < 1% striped), and Squire Valleevue Farm (SVF; 100% striped). The photograph displays the unstriped (left) and striped (right) color morphs

The Eastern Red-backed Salamander, *Plethodon cinereus*, is an ideal species for investigating mating patterns in a geographic context. This species is widespread throughout northeastern North America. It often displays a striped/unstriped color polymorphism that is genetically determined, while the frequency of morphs varies across the species’ range (Highton 1959; 2004; Moore and Ouellet 2014; Hantak et al. 2022). The ‘striped’ color morph exhibits a red stripe overlaid on a black dorsum and the ‘unstriped’ morph is completely black in dorsal coloration (Fig. 1). Much is known about the reproductive biology of these salamanders, including studies that have found that females exhibit parental care (Highton and Savage 1961; Ng and Wilbur 1995) and that both sexes have some degree of mate preference (Mathis 1991a; Walls et al. 1989). Further, *P. cinereus* is territorial and males and females defend sites with preferred microclimate conditions and high-quality (i.e., calorie-rich) dietary prey (Gabor and Jaeger 1995; Jaeger et al. 2016). Prior research has also found that the morphs demonstrate size and color assortative mating in at least one population (Anthony et al. 2008; Acord et al. 2013).

In this study, we examined mating interactions in *P. cinereus* across six populations that vary in color morph frequency from monomorphic striped to polymorphic to monomorphic unstriped (Fig. 1). Earlier work on *P. cinereus* has demonstrated geographic variability in sexual size dimorphism, with either females as the larger sex or no difference in size between the sexes (reviewed in Anthony and Pfingsten 2013). In populations with female-biased sexual size dimorphism, we predicted larger females, that are presumably more fecund (Nagel 1977; Lotter 1978), would be paired with larger males. We also predicted that larger females would be more often associated with larger males, as larger males have been shown to defend higher-quality territories (Verrell 1995; Eddy et al. 2016). Anthony et al. (2008) and Acord et al. (2013) both found evidence that the color morphs of *P. cinereus* demonstrate assortative mating by color in one Ohio population. These studies also found that smaller females paired more often with unstriped males, while larger females paired more often with striped males, which may be related to striped males holding higher-quality territories and consuming more profitable prey (Anthony et al. 2008, 2017; Reiter et al. 2014; Stuczka et al. 2016). Thus, in polymorphic populations, we predicted that morphs would demonstrate assortative mating by color morph and that larger females would be paired more often with striped males, while smaller females would be paired more often with unstriped males.

## Methods

In spring of 2014, we placed 100 porcelain tiles (30.5 cm^2^) each separated by three meters and laid out in a grid at six localities (*n* = 600 tiles total) in northern Ohio for a multi-year monitoring project. Such artificial cover objects provide repeatable, standardized, high-quality territories for small plethodontid salamanders (Mathis 1990; Monti et al. 2000). Northern Ohio is ideal for studying this color polymorphism due to the discovery of many populations that vary in morph frequency, including populations that have a high frequency of the unstriped morph, which is rare (Pfingsten and Walker 1978; Moore and Ouellet 2015; Hantak et al. 2015). We selected sites based on variation in color morph frequency: Squire Valleevue Farm (SVF; 100% striped), Chapin Forest Reservation (CF; 100% striped), Cuyahoga Valley National Park (CVNP; 80% striped), Edison Woods Reservation (EW; 45% striped), East Harbor State Park (EH; 8% striped), and the Heineman property on South Bass Island (SBI; < 1% striped; Fig. 1).

We surveyed each site once per week when feasible for presumptive mating pairs in the spring (March–June) and fall (September–November) of 2014–2016. Adult *P. cinereus* are territorial and will expel intruders from their cover objects, including unwanted individuals of the opposite sex (Mathis 1990, 1991b). Previous work has suggested that male–female pairs found under the same cover object within ~ 30 cm of each other have a high likelihood of being mating pairs (Gillette et al. 2000; Jaeger et al. 2002). As our artificial cover objects were 30.5 cm^2^, we assumed male–female pairs found together were a mating pair (Anthony et al. 2008). Sex was determined by the shape and size of salamander snouts: adult male *P. cinereus* in reproductive condition have an enlarged, broad snout, whereas in the remaining portion of the year they possess a more rounded snout (Anthony and Pfingsten 2013). Female *P. cinereus* always possess a rounded snout and can be identified by the presence of eggs, which are often visible through the ventral side of the body. However, in some cases, eggs are not visible in reproductively viable individuals; thus the mating status of every female in this study was not perfectly known. We assumed every female with a reproductive male constituted a mating pair. When a presumptive mating pair was found, we recorded sex, color morph (presence/absence of a dorsal stripe), snout-vent length (SVL), and mass (g). Prior to data analysis, we removed individuals from the dataset that were smaller than 32 mm SVL for males and 34 mm for females, which reflects sexual maturity data collected from the CVNP site (Anthony and Pfingsten 2013). A standardized photograph was taken of the unique ventral black and white mottling of each individual salamander to account for recaptures under artificial cover objects. For tests of sexual size dimorphism, we retained all adults regardless of pairing, but all recaptures were removed. For other analyses, if the same pair was repeatedly found under a cover object, we only retained data from the initial observation. However, individuals observed with a different partner in subsequent encounters were included in the dataset. Female *P. cinereus* produce a single clutch every one to two years, but store sperm and multiple paternity is likely common (Sayler 1966; Liebgold et al. 2006). Thus, documenting associations with multiple presumptive partners provides ecologically pertinent data on the species’ reproductive behavior.

### Statistical analyses

To determine whether *P. cinereus* demonstrates sexual size dimorphism, we ran an ANOVA to test the effects of site, sex, and their interaction on salamander SVL. To further examine population effects, we performed a separate ANOVA for each site with sex as the predictor variable and salamander SVL as the response variable.

To test for body size relationships between paired males and females, we used log-transformed values of salamander SVL. We kept each site separate for tests of mating interactions as each population is geographically separated (12 km–122 km), variable in color morph frequency, and differs in patterns of genetic variation (Hantak et al. 2019; Waldron et al. 2019, 2025b; Radomski et al. 2020). We then ran linear models using R’s base lm function (R Core Team 2024) to test for an association between paired male and female body size in all six populations. Models were run with female log-transformed SVL as the predictor variable and male log-transformed SVL as the response variable for each population.

To examine whether the distribution of color morph pairs differed within polymorphic populations (CVNP, EW), we ran G-tests of independence using the R package *DescTools* (Signorell 2024). To test for mate associations based on color morph and body size, we ran binomial logistic regressions using the R base glm function (R Core Team 2024). In all models, color morph was coded as 1 for striped and 0 for unstriped morphs. We ran separate models to examine the presumptive mate choice of both male and female color morphs and the predictor variables included the interaction between color morph and log transformed SVL for the opposite sex. For example, when male color morph was the response variable, we used the predictors of female color morph x female log transformed SVL, which was mean centered and scaled. Models initially contained covariates including year and season, but these were not statistically significant in most tests and were removed from the final analyses. We did not consider the EH site to be polymorphic due to the low number of striped morphs. All analyses were performed using R software V4.4.2 (R Core Team 2024).

## Results

**Fig. 2:**
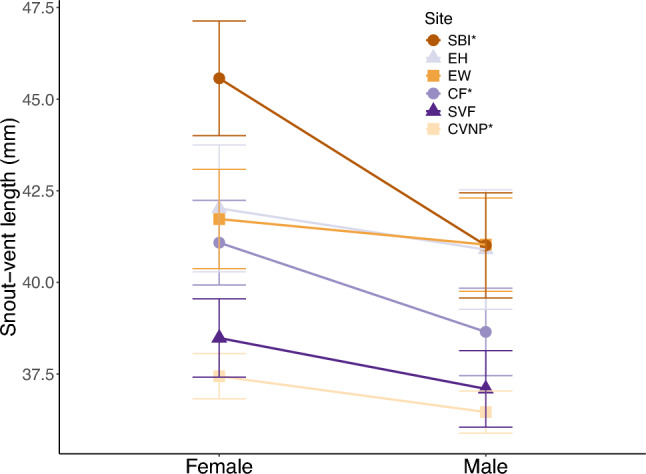
Sexual size dimorphism across the six study sites: Chapin Forest Reservation (CF; 100% striped), Cuyahoga Valley National Park (CVNP; 80% striped), East Harbor State Park (EH; 8% striped), Edison Woods Reservation (EW; 45% striped), South Bass Island (SBI; < 1% striped), and Squire Valleevue Farm (SVF; 100% striped). Populations in the legend with an asterisk (*) next to their names demonstrate significant patterns of sexual size dimorphism

Across six populations and six field seasons, we observed 172 independent male/female pairs. The number of pairs at each site was: 20 SVF; 17 CF; 58 CVNP; 34 EW; 25 EH; and 18 SBI. We found a significant effect of sex (F_1,330_ = 20.25, *p* < 0.001), site (F_5,330_ = 37.96, *p* < 0.001), and the interaction between sex and site (F_5,330_ = 2.33, *p* = 0.042) on salamander SVL. A Tukey HSD post hoc test revealed that salamanders (males and females combined) at CVNP and SVF are, on average, similar in size, but are smaller than all other sites (Supplemental Table 1). Male and female SBI salamanders are larger than individuals from eastern localities (CVNP, Squire, and Chapin; Fig. 2, Supplemental Table 1). ANOVA models from individual sites indicate that females are larger than males at three of the six sites: CF (F_1,31_ = 8.93, *p* = 0.005), CVNP (F_1,114_ = 5.27, *p* = 0.024); and SBI (F_1,33_ = 19.07, *p* < 0.001; Fig. 2). There was a trend for larger females at SVF (F_1,39_ = 3.52, *p* = 0.068; Fig. 2). No sexual size dimorphism was found at EH (F_1,49_ = 0.90, *p* = 0.347) or EW (F_1,64_ = 0.56, *p* = 0.456; Fig. 2).

**Fig. 3:**
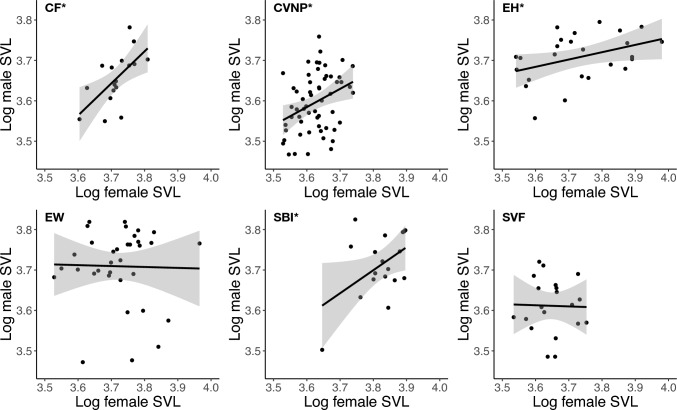
Paired female and male body size (snout-vent length) regressions for each population: Chapin Forest Reservation (CF; 100% striped), Cuyahoga Valley National Park (CVNP; 80% striped), East Harbor State Park (EH; 8% striped), Edison Woods Reservation (EW; 45% striped), South Bass Island (SBI; < 1% striped), and Squire Valleevue Farm (SVF; 100% striped). Populations with an asterisk (*) next to their name possess a significant association

We found a positive association between paired male and female body size at CF (*β* = 0.79, SE = 0.26, *p* = 0.008), CVNP (β = 0.45, SE = 0.16, *p* = 0.008), EH (β = 0.18, SE = 0.09, *p* = 0.048), and SBI (β = 0.57, SE = 0.27, *p* = 0.047; Fig. 3). No body size relationship between presumptive mating pairs was found at EW (*β* = − 0.02, SE = 0.18, *p* = 0.898) or SVF (β = −0.03, SE = 0.27, *p* = 0.918; Fig. 3).

In polymorphic sites, we found no evidence for morph-based pairings when considering color alone: CVNP (*G* = 0.38; *p* = 0.538) or EW (*G* = 0.17; *p* = 0.681; Supplemental Fig. 1). We tested for color morph and body size mate associations for both males and females at the polymorphic sites CVNP and EW. We found no relationship between male or female color and body size (SVL) at either of these sites (Table 1).

**Table 1:** Fixed effect estimates of color morph by body size mate associations across the two polymorphic sites: Cuyahoga Valley National Park (CVNP; a,b) and Edison Woods Reservation (EW; c,d)

Term	Estimate	Std. error	*p* value
(a) CVNP: male color morph ~ female color morph x female body size			
Intercept	7.446	4.557	0.102
Female SVL (log)	6.675	4.715	0.157
Female color morph	− 5.528	4.609	0.230
Female SVL (log): female color morph	− 6.345	4.777	0.184
(b) CVNP: female color morph ~ male color morph x male body size			
Intercept	20.60	17.92	0.250
Male SVL (log)	13.78	11.93	0.248
Male color morph	− 19.56	17.93	0.275
Male SVL (log): male color morph	− 14.36	11.94	0.229
(c) EW: male color morph ~ female color morph x female body size			
Intercept	− 0.681	0.598	0.255
Female SVL (log)	0.330	0.525	0.530
Female color morph	0.407	0.773	0.598
Female SVL (log): female color morph	− 0.009	0.811	0.991
(d) EW: female color morph ~ male color morph x male body size			
Intercept	− 0.121	0.588	0.837
Male SVL (log)	0.162	0.507	0.750
Male color morph	0.095	0.874	0.913
Male SVL (log): male color morph	0.601	0.769	0.434

Other sites were not included in this analysis as they were monomorphic or nearly monomorphic

## Discussion

Assortative mating is a mechanism by which phenotypic variation can be maintained and distinct forms can, given time and the proper conditions, diverge into separate species (West-Eberhard 1986; Kirkpatrick and Ravigne 2002; Bolnick and Kirkpatrick 2012). In this study, we examined presumptive mating pairs of *P. cinereus*, as inferred from male–female associations under cover objects, across six populations that varied in color morph frequency. We expected that all populations would reveal female-biased sexual size dimorphism, and that larger females would be paired with larger males. Based on previous work, we also predicted morphs would exhibit assortative mating by color and that striped males would pair more often with larger females (Anthony et al. 2008). We documented female-biased sexual size dimorphism in three of six study sites and inconsistent patterns of apparent mate association based on body size and color morph across populations. We found evidence that larger females pair with larger males in four of six populations. Further, we found no evidence for presumptive mate associations based on color and body size in polymorphic populations.

Body size and sexual size dimorphism in *P. cinereus* varied among the populations we sampled. Larger salamanders were found at sites further west and generally closer to Lake Erie. Sites closer to Lake Erie experience warmer temperatures during the winter months, and areas in the middle and western portions of the state have a lower annual mean precipitation (Armitage and Lipps 2013). In addition, past work at SBI, an island where the largest salamanders were present, found that climate varies from that of the mainland, with comparatively higher temperatures in the summer and fall, lower temperatures in the winter and spring, lower annual precipitation, and longer frost-free periods (Verber 1955). A few studies have examined body size variation in *P. cinereus* in relation to climate, but findings have been discordant, particularly regarding the influence of temperature on the direction of body size change (Caruso et al. 2014; McCarthy et al. 2017; Hantak et al. 2021); however, only one of these studies took morphs into account (Hantak et al. 2021).

Female-biased sexual size dimorphism was found at three study sites (CF, CVNP, and SBI), while there was no difference in size between sexes at the remaining three populations. Many studies have shown sexual size dimorphism varies over the range of vertebrate taxa (e.g., Pearson et al. 2002; Cox and Calsbeek 2010; Litzgus and Smith 2010; DeGregorio et al. 2018). Patterns of sexual size dimorphism within the salamander family Plethodontidae are also variable (Staub 2021), but female-biased size dimorphism is more common (Bruce 2000; Kupfer 2007; Pincheira-Donoso et al. 2021). Few studies have investigated geographic variation in sexual size dimorphism in *Plethodon* (but see Bruce 2000); however, Leclair et al. (2006) found a greater difference in female-biased sexual size dimorphism for *P. cinereus* in the northern part of its range (e.g., Canada and Michigan) compared to more southernly study sites (e.g., Ohio, Maryland, Virginia). Several studies have suggested that female-biased sexual size dimorphism in amphibians should increase with higher latitudes, where environments are more seasonal, because larger body size paired with larger clutch or egg size may be advantageous as the number of opportunities to reproduce is lower (Morrison and Hero 2003; Nali et al. 2014). Our results do not indicate any latitudinal signal, although our furthest sites only spanned a latitudinal distance of approximately 47 km. Yet, as the study sites are disjunct and genetic differentiation across populations is substantial (Hantak et al. 2019), it is probable that local, unmeasured selective pressures are driving variation in sexual size dimorphism across our study sites (Blanckenhorn et al. 2006). However, in addition to selection pressures, demographic and life history variation may also contribute to differences in sexual size dimorphism across populations. For example, sex differences in age at sexual maturity (i.e., sexual bimaturation) could result in spatial variation in sexual size dimorphism in *P. cinereus* (Stamps 1993; Stamps and Krishnan 1997).

A positive body size relationship between paired males and females was found in one monomorphic striped population (CF), one monomorphic unstriped population (SBI; < 1% striped), one polymorphic population (CVNP; 80% striped), and one polymorphic population that has a high frequency of the unstriped morph (EH; 92% unstriped). Thus, there does not appear to be a link between color morph frequency and male–female size associations in *P. cinereus*. Spatial variation in the size of paired males and females is likely the consequence of divergent selective pressures across our study extent (Moura et al. 2021). Geographic variation in presumptive mate size associations may be a mechanism that drives reproductive isolation across populations, but other evolutionary mechanisms, such as gene flow from populations experiencing divergent selective pressures, can counteract these effects (Servedio 2016). Hantak et al. (2019) found low levels of genetic differentiation between populations in eastern Ohio. For instance, SVF (site 25 in Hantak et al. 2019) and CF (site 27) are separated by F_ST_ = 0.03 (distance of 11.6 km). In contrast, sites in the western portion of Ohio were more geographically and genetically separated from other sites. For example, EH (site 2) is separated from sites near the EW population by F_ST_ ≥ 0.25 (sites 2 and 4; 22.5 km). Therefore, the combination of these factors, including size-related pairings, reduced gene flow, and increased genetic drift, may have important evolutionary consequences.

A central goal of our work was to determine whether the striped and unstriped morphs of *P. cinereus* demonstrate apparent color-assortative mating across multiple populations. In contrast to previous studies (Anthony et al. 2008; Acord et al. 2013), we found no evidence of assortative mating by color morph alone, even though one of our study sites (CVNP; 80% striped) was identical to past work. Because our study was focused on spatial trends, by necessity less time was spent at each individual site. Therefore, the discrepancy between the current work and previous research at CVNP may relate to sample size differences. To examine statistical power estimates across studies, we used the R package *pwr* (Champely et al. 2018). In Anthony et al. (2008) and Acord et al. (2013), we found low statistical power (*N* = 94, ES = 0.17, power = 0.39; *N* = 112, ES = 0.16, power = 0.39, respectively). Using the mean effect size calculated from the aforementioned studies, the statistical power in our study was moderately lower (*N* = 58, ES = 0.165, power = 0.24). However, when combining data from all three studies at CVNP, we find stronger statistical power to detect trends (*N* = 264, ES = 0.165, power = 0.77), and strong evidence for presumptive color assortative mating (*G* = 7.26; *p* = 0.007). Although sample sizes could be combined for this well-studied site, detecting color assortative mating within multiple populations will remain challenging as a substantial amount of data is required. Indeed, the sample size was lower in the other color polymorphic site (EW *N* = 34); thus, it is difficult to interpret whether our non-significant results are representative of random mating at these sites. On the other hand, assortative mating must be strong and consistent to overwhelm the powerful homogenizing influence of interbreeding between the morphs, and the sample sizes in our study were sufficient to detect high levels of assortative mating. Overall, our study indicates that assortative mating by color morph is not strong or consistent enough to result in sympatric species formation.

Previous research on assortative mating in *P. cinereus* at CVNP also found that larger females were paired more often with striped males (Anthony et al. 2008; Acord et al. 2013). The authors suggested this finding may be due to striped males consuming more energetically profitable prey and holding higher-quality territories compared to unstriped morphs (Anthony et al. 2008; Reiter et al. 2014; Stuczka et al. 2016). As with other aspects of this study, we found geographic variation in male–female interactions. The polymorphic CVNP (80% striped) and EW (45% striped) sites showed no evidence of color morph mate association when considering both body size (SVL) and color morphology. Thus, there is a discrepancy between our study and previous work at the CVNP site (Anthony et al. 2008; Acord et al. 2013). This disparity may be due to the different measures of body size used across studies. Indeed, in our study we used SVL to examine paired male–female associations, but previous work used mass-length residuals to examine salamander body condition (Anthony et al. 2008; Acord et al. 2013). To account for this difference, we conducted a post hoc analysis to examine the relationship between paired males and females based on color and body size at CVNP using body size residuals (from an ordinary least squares regression of mass and SVL) to match previous work. Here, we detected a trend in which striped males were paired with larger unstriped females (interaction term: *β* = − 1.78, SE = 0.94, *p* = 0.060). While we expected striped males would pair more often with larger females—as larger female size is often associated with higher fecundity (i.e., fecundity selection; Pincheira-Donoso and Hunt 2017), including in *P. cinereus* (Jaworski et al. 2018)—the association with unstriped morphs was not anticipated. Most studies focused on morph differences have documented that the striped morph has a better diet (Anthony et al. 2008; Stuczka et al. 2016; but see Hantak et al. 2020), higher levels of territorial behavior (Reiter et al. 2014), and lower rates of disease prevalence (Venesky et al. 2015). In addition, several studies have documented increased rates of predation on the unstriped morph compared to the striped morph (Moreno 1989; Venesky and Anthony 2007; Otaibi et al. 2017). However, Grant et al. (2018) found higher rates of predation and lower survival estimates for the striped morph in a Maryland population. While the trends we found here lack a clear pattern, it is likely that the co-adapted traits associated with morphs are largely influenced by selection pressures that vary widely over spatial and temporal scales (Gosden and Svensson 2008; Calsbeek et al. 2012; Hantak and Kuchta 2018).

Uncovering geographic variation in mating interactions across our study sites highlights the importance of integrating a spatial framework into research. As always, studies aimed at investigating traits such as mate associations will need to navigate the trade-offs between the number of sites and the sample size at each site. In our study, spatial variation was prominent, even though we focused on a relatively small portion of the species’ range. Morph alone did not strongly influence paired male–female associations across our study sites, suggesting that other mechanisms, such as negative frequency dependent selection, gene flow among populations, or niche partitioning are likely to also contribute to the maintenance of the striped/unstriped polymorphism in *P. cinereus* (Gray and McKinnon 2007; McLean and Stuart-Fox 2014; Hantak et al. 2019). As selection pressures are known to vary across spatial and temporal scales (Siepielski et al. 2009; Calsbeek et al. 2012), obtaining better data on the agents of selection and the traits under direct selection will aid in our understanding of the maintenance of this polymorphism within and across populations.

## Supplementary Information

Below is the link to the electronic supplementary material.

Supplementary file1 (DOCX 73 KB)

## Data Availability

The data produced from this work are available from the corresponding author upon request.
