# Supplementary material for: Spatial variation in sexual size dimorphism and mating associations in the color polymorphic Eastern Red-Backed Salamander (Plethodon cinereus)
Source: Oecologia. 2025 Nov 5;207(12):184. doi: 10.1007/s00442-025-05826-w (PMC12589318; doi:10.1007/s00442-025-05826-w)
Supplement: Supplementary file 1 — Supplementary file1 (DOCX 73 KB) [file 442_2025_5826_MOESM1_ESM.docx]

**Spatial variation in sexual size dimorphism and mating associations in the color polymorphic Eastern Red-backed Salamander (*Plethodon cinereus*)**

Maggie M. Hantak^1*^, Olivia L. Brooks^2^, Kyle M. Brooks^3^, Carl D. Anthony^4^, Cari-Ann M. Hickerson^4^, Kelly A. Williams^3^, Shawn R. Kuchta^3^

^1^Department of Biology, University of Dayton, Dayton, OH 45469, USA

^2^Department of Biological Sciences, Virginia Polytechnic Institute and State University, Blacksburg, VA 24061, USA

^3^ Department of Biological Sciences, Ohio Center for Ecological and Evolutionary Studies, Ohio University, Athens, OH 45701, USA

^4^Department of Biology, John Carroll University, University Heights, OH 44118, USA

*Correspondence: mhantak1@udayton.edu

Supplemental Table 1. Tukey’s post hoc comparisons of body size variation in *Plethodon* *cinereus* across study sites: Cuyahoga Valley National Park (CVNP), Chapin Forest Reservation (CF), East Harbor State Park (EH), Edison Woods Reservation (EW), South Bass Island (SBI), and Squire Valleevue Farm (SVF).

| **Site** | **Site** | **Difference** | **95% CI** | **p value** |
| --- | --- | --- | --- | --- |
| **CVNP** | **CF** | **-2.91** | **(-4.66, -1.17)** | **<0.001** |
| EH | CF | 1.59 | (-0.39, 3.56) | 0.196 |
| EW | CF | 1.52 | (-0.36, 3.41) | 0.191 |
| **SBI** | **CF** | **3.28** | **(1.13, 5.42)** | **<0.001** |
| **SVF** | **CF** | **-2.09** | **(-4.16, -0.02)** | **0.046** |
| **EH** | **CVNP** | **4.50** | **(3.01, 5.99)** | **<0.001** |
| **EW** | **CVNP** | **4.44** | **(3.07, 5.80)** | **<0.001** |
| **SBI** | **CVNP** | **6.19** | **(4.48, 7.90)** | **<0.001** |
| SVF | CVNP | 0.82 | (-0.79, 2.43) | 0.687 |
| EW | EH | -0.07 | (-1.71, 1.58) | 1.000 |
| SBI | EH | 1.69 | (-0.25, 3.63) | 0.129 |
| **SVF** | **EH** | **-3.68** | **(-5.54, -1.82)** | **<0.001** |
| SBI | EW | 1.75 | (-0.10, 3.60) | 0.074 |
| **SVF** | **EW** | **-3.61** | **(-5.37, -1.85)** | **<0.001** |
| **SVF** | **SBI** | **-5.37** | **(-7.40, -3.33)** | **<0.001** |

Supplemental Figure 1. Expected and observed proportions of presumptive mating pairs based on color morphology for polymorphic sites: Cuyahoga Valley National Park (CVNP; 80% striped) and Edison Woods Reservation (EW; 45% striped). The pie under each site name represents the estimated frequency of the striped (red) and unstriped (black) morphs at that population.
